# Combinational immune-cell therapy of natural killer cells and sorafenib for advanced hepatocellular carcinoma: a review

**DOI:** 10.1186/s12935-018-0624-x

**Published:** 2018-09-10

**Authors:** Faezeh Hosseinzadeh, Javad Verdi, Jafar Ai, Saieh Hajighasemlou, Iman Seyhoun, Frzad Parvizpour, Fatemeh Hosseinzadeh, Abolfazl Iranikhah, Sadegh Shirian

**Affiliations:** 1 Department of Tissue Engineering and Applied Cell Sciences, School of Advanced Technologies in Medicine, Tehran University of Medical Sciences, Tehran, Iran; 2 Iran Food and Drug Administration, Tehran, Iran; 3 Dentistry Faculty, Tehran University of Medical Sciences, Tehran, Iran; 4 Department of Gastroenterology, Faculty of Medicine, Qom University of Medical Sciences, Qom, Iran; 5 Department of Pathology, School of Veterinary Medicine, Shahrekord University, Shahrekord, Iran; 6 Shiraz Molecular Pathology Research Center, Dr. Daneshbod Lab, Shiraz, Iran

**Keywords:** Natural killer (NK) cells, Sorafenib, Hepatocellular carcinoma (HCC)

## Abstract

**Background:**

High prevalence of hepatocellular carcinoma (HCC) and typically poor prognosis of this disease that lead to late stage diagnosis when potentially curative therapies are least effective; therefore, development of an effective and systematic treatment is an urgent requirement.

**Main body:**

In this review, several current treatments for HCC patients and their advantages or disadvantages were summarized. Moreover, various recent preclinical and clinical studies about the performances of “two efficient agents, sorafenib or natural killer (NK) cells”, against HCC cells were investigated. In addition, the focus this review was on the chemo-immunotherapy approach, correlation between sorafenib and NK cells and their effects on the performance of each other for better suppression of HCC.

**Conclusion:**

It was concluded that combinational therapy with sorafenib and NK cells might improve the outcome of applied therapeutic approaches for HCC patients. Finally, it was also concluded that interaction between sorafenib and NK cells is dose and time dependent, therefore, a careful dose and time optimizing is necessary for development of a combinational immune-cell therapy.

## Background

Hepatocellular carcinoma is the fifth most common malignant tumor and second cause of cancer related death worldwide [1]. Despite of several attempts to improve the treatment options of this cancer, such as chemotherapy, loco regional ablation, surgical resection, intervene therapy or liver transplantation, only early-stage tumors can be treated, while this disease often diagnosed at an advanced stage [2]. Therapeutic approaches used to treat HCC patients are selected based on the stage of the tumor [3]. Approximately, 40% of HCC patients diagnosed at early stages of the disease are good candidates for curative treatment. Patients with advanced HCC have an average survival rate of less than 1 year and can be divided into three groups; intermediate-stage disease (stage B), advanced-stage disease (stage C) and end-stage disease (stage D) [4]. Liver resection is the first choice for very early-stage HCC and non-cirrhotic patients who consist the minority of patients [5]. Liver transplantation has a better outcome for early-stage HCC patient. The advantage of liver transplantation is that the tumor and underlying cirrhosis have been removed and the risk of HCC recurrence is minimized. For early-stage HCC patients who are not qualified for liver resection or transplantation, other less invasive therapies, such as percutaneous treatments or radiofrequency ablation, are the appropriate alternatives. Furthermore, transarterial chemoembolization may be suitable therapy for intermediate-stage HCC patients (approximately 20% of HCC patients) which prolongs survival rate from 16 months to 19–20 months [3, 6]. These curative treatments increase the chance of approximately 5-year survival rates up to 75% [6]. Since the number of liver donors are limited and due to advanced stage of HCC or hepatic dysfunction, less than 20% of HCC patients are qualified for such treatments [7, 8]. Sorafenib is the first-line drug that has been approved for treatment of end stage patients with advanced or metastatic HCC who have median survival duration of 3–4 months [3, 6, 9, 10]. In spite of the survival benefit of each treatment for HCC patient, therapeutic options for advanced HCC patient are limited and their median survival rate for these patients are less than 1 year [6]. Therefore, developing new systemic therapies is urgently needed for this aggressive disease. Cancer immunotherapy highly considered in the last decades and is growing in preclinical and clinical phases of HCC treatment [11–13]. There are many immunotherapeutic approaches for treatment of advanced HCC patients, including: several vaccines, molecularly targeted drugs such as sorafenib, passive immunotherapy such as adaptive transfer of immune cells or immune modulatory reagents and combinational therapy [11]. The focus of the present review was on NK cell based immunotherapy (its advantages and dysfunctions) and its correlation with sorafenib (chemo immunotherapy) for treatment of HCC patients, as well as investigating the combinational therapy approach and mechanisms underlying the effects of NK cell and sorafenib on each other’s performance.

## Sorafenib

**Fig. 1:**
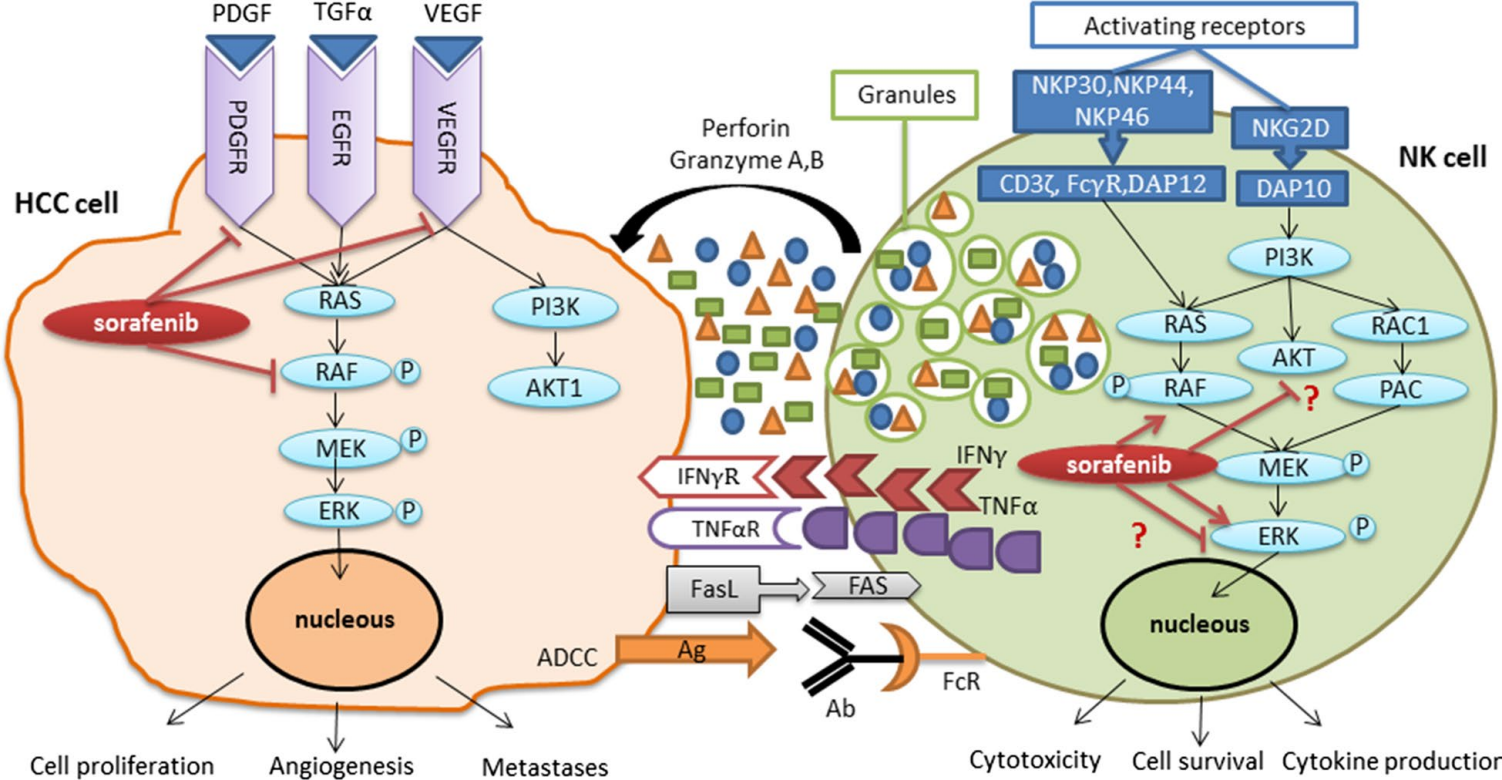
The signaling pathways of sorafenib effect on the HCC tumor progression and NK cell effector function against tumor cells

Sorafenib which is the first FDA approved drug for treatment of HCC, is a multi-kinase inhibitor that can block proliferation and angiogenesis of tumor cell by inhibiting a wide range of molecular targets including serine/threonine kinases, receptor tyrosine kinases, rapidly accelerated fibro sarcoma (Raf) kinases, vascular endothelial growth factor receptor 2, 3 (VEGFR2, VEGFR3), platelet-derived growth factor receptor (PDGFR), FLT3, Ret, and c-KIT [14, 15] (Fig. 1). Although phase III clinical trials of sorafenib in advanced HCC patients resulted in improved overall survival rate and delayed tumor progression, but only a 2–3% overall response rate of potent antiangiogenic effect of sorafenib was detected in clinical treatment of HCC [16, 17]. Furthermore, around 2–3% of tumor regression and usually less than 1 year survival rate are observed in clinical phase of HCC treatment applying sorafenib. In addition, lower dose of sorafenib is often needed since administration of full dose of sorafenib (800 mg/day) leads to some adverse drug reactions which prevent continuing the therapy [18, 19]. Although systemic treatment with sorafenib is a useful therapeutic approach for HCC patients, its effect on survival rate is still limited since HCC cells are complex and heterogeneous cells with improper activation of several signaling pathways [20, 21]. Therefore, these unsatisfactory factors illustrate the urgent need for developing a combinational therapy with sorafenib plus chemotherapy or other targeted therapeutic agents in order to increase the performance of sorafenib and better suppression of HCC.

### Combinational therapy of HCC with sorafenib and other agents

Over the recent years, to improve the efficacy of sorefenib based treatment of HCC patients, the combination therapy strategies has been proposed and several studies have investigated about these coupled treatments. The combination of trans-catheter arterial chemoembolization (TACE) and sorafenib in patients with intermediate-stage HCC is well tolerated and efficacious [22, 23]. Among patients with advanced HCC, combination of sorafenib and doxorubicin improved the progression free survival and overall survival rate of patients compared with doxorubicin mono-therapy [24]. As reviewed by Abdel-Rahman [25], 8 trials involving 272 HCC patients treated with a combination of sorafenib and couple of anticancer agents (especially mTOR inhibitors) resulted in a more effective and tolerable treatment. The combination therapy using rapamycin and sorafenib, in an orthotopic xenograft model of human HCC illustrated that compositional treatment enhanced the anti-tumor activation against HCC cells compared with single treatment administering either rapamycin or sorafenib; furthermore, a significant inhibition of tumor cell proliferation was observed as a result of this combination treatment [26]. Combinational immunotherapy using sorafenib is a very novel approach applied for treatment of HCC patients. Therapeutic efficacy of sorafenib and anti-programmed death-ligand 1 (PD-L1) monoclonal antibody (mAb) has been shown to result in a considerable reduction of tumor growth by induction of effective NK cell responses against HCC [27]. As a result of in vitro and in vivo experiments on HCC patients treated with the combination of sorafenib and erlotinib (inhibitor of epidermal growth factor receptor (EGFR) tyrosine kinase), no additional effect was observed and the survival rate of patients with advanced HCC did not improve [28, 29]. Co-administration of sorafenib and mammalian target of rapamycin (mTOR) inhibitor could be an effective approach to prevent recurrent HCC after liver transplantation; however, their toxicity and efficacy need to be further evaluated [30]. In the other study, combination of sorafenib and long-acting octreotide was showed to be an active and well tolerable therapy for HCC patients [31]. On the other hand, metronomic chemotherapy with tegafur/uracil combined with sorafenib resulted in an introductory activity to modest improvement of sorafenib efficacy in advanced HCC patients [32]. SC-43 is a sorafenib derivative with more anti HCC activity than sorafenib which induces apoptosis in sorafenib-resistant HCC cells. In a recent study conducted by Chao et al. [33], combination of sorfenib with SC-43 to treat HCC patients resulted in decrease p-STAT3 signaling and tumor size and prolonged the survival rate of murine HCC models. Co-administration of TLR3 agonist (lysine-stabilized polyinosinic polycytidylic-acid [poly-ICLC]) with sorafenib could controls HCC progression in vivo and promoted immune activation particularly local NK cells, T cells, macrophages and dendritic cells. It could also increase apoptosis and reduced proliferation of HCC cell lines in vitro, via impairing phosphorylation of AKT, MEK and ERK [34]. Furthermore, inhibition of aberrant mesenchymal-epithelial transition factor (c-Met) activation has been found to be a target for cancer therapy. DE605 (a novel c-Met inhibitor) together with sorafenib synergistically induced apoptosis in HCC cells via activation of FGFR3/ERK pathway [35].

### NK cells

Natural killer cells were identified in 1975 as a subset of lymphocytes with cytoplasmic granules that contribute to the first line of innate immunity in the control of viral infections. The highest frequency of NK cells (CD56^+^, CD3^−^) is in lung, liver, peripheral blood, spleen, bone marrow, lymph nodes, and thymus. The function of NK cells is very tissue microenvironment dependent; especially in a healthy liver that cytotoxicity and cytokine production of NK cells is higher than that of peripheral NK cells. Furthermore, the proportion of NK cells in the liver (30–50%) is more than peripheral blood NK cells (5–20%), in humans. It has been suggested that progenitors of circulating NK cell in peripheral blood migrate into the liver and differentiate into hepatic NK cells with distinctive properties, such as higher level of cytotoxicity, expression of cytotoxic mediators and cytokine production. There are two subtypes of NK cells, including CD56 bright and CD56 dim subsets, which are categorized based on the levels of CD56 expression. CD56 bright NK cells are induced from NK cell precursors and probably differentiate into CD56 dim NK cells (Approximately 90% of circulating NK cells and 50% of liver NK cells). Moreover, CD56 dim NK cells are more cytotoxic against target cells by releasing lower amounts of cytokines than CD56 bright NK cells [36–38].

### Regulation of NK cell activation and its interaction with other cells

As reviewed in recent papers, the function of human NK cells is regulated by several complex receptors, including inhibitory receptors, activating receptors and cytokine receptors that would bind to their ligands on the surface of the target cells and various cytokines that are secreted from diverse cells into the environment [39, 40]. Activation of NK cells is correlated to other immune cells, including dendritic cells, macrophages, T cells and endothelial cells via production of various cytokines [41], for instance, activated NK cells can promote maturation of dendritic cells and differentiation of immature helper T cells [42, 43]. On the other hand, activation of NK cells can be modulated by other immune cells via production of activator (IFN-α/β, IL-2, IL-12, IL-15, IL-18, and IFN-γ) or inhibitors (transforming growth factor beta (TGF-β) and IL-10) [38]. For example, activated Kupffer cells (KCs) can promote the activation of NK cells by Toll-like receptor (TLR) ligand through a cell-to-cell contact or production of IL-12 and IL-18 [44, 45]. Moreover, dendritic cells can promote the activation of NK cells which may lead to a massive degeneration of hepatocytes through Fas/FasL interactions in a mouse model of HBV infection [46]. In contrast, T-regulatory cells (Tregs) can trigger the suppression of NK cell by production of IL-10 and TGF-β [47].

The surface molecules expressed on the target cells determine the function of NK cells via interaction with diverse NK cell receptors. One of the biggest advantages of NK cells, that has attracted the attention of many researchers to this field of immune cell therapy, is the reduced activity of NK cell in the presence of surface major histocompatibility complex (MHC) class I genes that expressed in normal cells and often down regulated or silent in tumor cells to evade recognition by anti-tumor T cells. These events are known as ‘missing-self’ model and it has been shown the absence of MHC-I expression can be detected by NK cell-inhibiting receptors which result ininhibition of NK cells and prevention of deleterious graft versus host disease (GVHD) event. On the other hand, the activating receptors of NK cells are responsible for identification of induced ligand from DNA damage or cellular stress on the surface of the tumor cells, which lead to cytotoxic activity of NK cells and providing graft vs. leukemia (GvL) effect [48–50]. As reviewed in recent articles, NK cells can attack tumor cells not only directly via several mechanisms, including secreting cytoplasmic granules (such as perforin and granzymes), death receptor-mediated apoptosis (such as FasL or TNF-related apoptosis-inducing ligand (TRAIL)), releasing various cytokines (such as IFN-γ) or through antibody dependent cellular cytotoxicity (ADCC) by expressing CD16 antigen on the surface of NK cells (Fig. 1), but they also act indirectly through interaction with other immune cells via production of different cytokines, chemokines and growth factors [41]. Furthermore, various experimental, pre-clinical and clinical studies have been performed in order to improve the treatment of various cancers via cell therapy with NK cells [41, 51].

### Hepatic NK cells in the pathogenesis of hepatocellular carcinoma; advantages, disadvantages and dysfunctions

NK cells are the essential components of innate immune system in the liver. They are involved in hepatic immune defense and pathology and perform both the protective and detrimental functions in human liver diseases. Some beneficial functions of these cells are inhibition of viral infection, prevention of liver fibrosis, and cytolytic activity against hepatic stellate cells as well as HCC cells in order to inhibit liver fibrosis and tumor growth, respectively. However, some detrimental roles have also been reported for NK cells, including hepatocellular damage and inhibition of liver regeneration [38].

Inhibition of the frequency or functional impairment of NK cells is correlated with the progression and metastasis of various tumors in human and animal models [52, 53]. It has been reported that the number of peripheral and intrahepatic NK cells were significantly reduced in patients at various stages of HCC compared to that in healthy individuals [54, 55]. Furthermore, the reduction of NK cells proportion is noticeable in the advanced stages of HCC, mainly reflected in tumor-infiltrating NK cells [56, 57]. Although reposition of functional NK cells in hepatic tissues of HCC patients could improve survival rate of them [58], often reduced infiltration and impaired functional activities, including TNF-α and IFN-γ production as well as releasing cytoplasmic granules (i.e., granzyme A, granzyme B and perforin), could be observed in advanced HCC patients and might be associated with the progression and invasion of HCC [54]. The significant reduction of CD56 dim NK subsets in peripheral blood and in the tumor regions compared to non-tumor regions in the HCC patients with reduced levels of IFN-γ production and cytotoxic activity indicate the suppressed tumor-surveillance functions of NK cells in advanced HCC patients [54, 59–61]. Dysfunction of NK cells at advanced stages of HCC is triggered by myeloid-derived suppressor cells (MDSCs), monocytes/macrophages and fibroblasts via NKp30 receptor, CD48/2B4 interactions or PGE2 and IDO, respectively [57, 59, 62]. Major histocompatibility complex (MHC) class I–related chain A (MICA), which is highly expressed on the surface of human HCC, is the ligand of NKG2D (activating receptor of NK cells). Soluble form of MICA (sMICA), which is released from cancer cells in order to escape from immune system, is an antagonist of MICA/NKG2D pathway [58]. It has been illustrated that serum levels of sMICA are increased in patients with advanced HCC which in turn result in dysfunction or exhaustion of NK cells [60].

### Development of NK cell based immunotherapy for HCC disease

Despite the critical role of NK cells in the hepatic regeneration during viral infection, hepatic fibrosis and hepatocellular carcinoma, the frequency and cytolytic activity of NK cells have been impaired over the progression of liver diseases [63]. Therefore, as summarized in recent articles [40, 63], various strategies have been used to overcome dysfunction or exhaustion of NK cells as a reliable therapeutic strategy for HCC treatment. The main approaches which have been studied in order to improve NK cell-based immunotherapy against HCC including chemo-immunotherapy, NK cell transplantation, gene modified NK cell lines, genetic manipulation, cytokine therapy and mAb therapy in the preclinical phase are reviewed in Table 1. Before starting clinical trials, extensive pre-clinical studies is necessary which involve in vitro and in vivo experiments to obtain preliminary efficacy, toxicity and signaling pathway information of one special cell therapy. These preclinical studies can help tp researchers to decide whether a cell based therapeutic approach has scientific merit for further development as an investigational new treatment.

**Table 1:** Preclinical (in vitro and in vivo) studies of NK cell based immunotherapy for HCC

Interventions	Design	Strategy	References
Activation of NK cells by intrahepatic injection of α-galactosylceramide- pulsed dendritic cells	Mouse model of liver tumor (in vivo)	Chemical therapy and DC cell transfer	[64]
Activation of NK cells by Adenoviral IL-12 gene transfer	Rats model of orthotopic HCC (in vivo)	Cytokine gene therapy	[64]
Activation of innate and acquired immunity by injection of IL-12gene-transduced dendritic cells	Mouse model of liver tumor (in vivo)	Cytokine gene therapy and DC cell transfer	[65]
Augmentation of anti-human HCC effect of NK cells by IL-15 gene- modified NKL cell line	Xenograft tumor models (in vivo)	Cytokine gene modified NK cell lines and NK cell transfer	[66]
Enhancement of anti-human HCC function of NK cell line by hIFN-alpha gene modification	Cytotoxic assay (in vitro) and Xenograft tumor models (in vivo)	Cytokine gene modified NK cell lines and NK cell transfer	[67]
Strong role of MICA in HCV-related HCC patients	Genome-wide association study (in vitro)	Gene therapy	[68]
Activation of NK cells via binding of MICA and NKG2D	Cytotoxic assay (in vitro)	Gene therapy and MAb therapy	[69, 70]
Anti-HCC drugs such as sorafenib triggered anti-tumor activity of NK cells by down-regulation of ADAM10 or ADAM9 and increasing membrane bound of MICA on surface of the tumor cells	Cytotoxic assay (in vitro)	Chemo-immunotherapy	[71, 72]
Sorafenib induced antitumor activity of NK cells by modulating the crosstalk between tumor-associated macrophages (TAM) and NK cells	Animal experiments (in vivo) and killing assay (in vitro)	Chemo-immunotherapy	[73]
Antitumor effects of bortezomib via induction of MICA/B expression on HCC cells and increasing cytolytic activity of NK cells	Cytotoxicity assay (in vitro)	Chemo-immunotherapy	[74]
Over-expression of MICA or MICB on hepatoma cells and activation of NK cells via induction of NKG2D ligands by histone deacetylase inhibitor	Cytotoxicity assay and epigenetic study (in vitro)	Chemo-immunotherapy	[75–77]
Ex vivo generation, activation and expansion of NK cells for immunotherapy of advanced cancer	(In vitro and in vivo)	NK cell transfer	[41, 78–80]
Increasing proliferation, survival and anti-tumor activation of human NK cells by interleukin-15 gene modification	Cell culture and cytotoxicity assay (in vitro)	Cytokine gene modified NK cell lines	[81, 82]
Prevention of relapse of HCC relapse after partial hepatectomy by adoptive transfer of TRAIL-expressing NK cells	Murine HCC metastasis model (in vivo)	Adoptive transfer of activated NK cells	[83]
Rapid and sustained regression of HCC by adoptive transfer of allogeneic suicide gene-modified killer cells mainly NK cells	Cell culture and animal models (in vitro and in vivo)	Adoptive transfer of gene-modified killer cells	[84]
Induction of proliferation and activation of NK cells as well as inhibition of tumor growth, neovascularization and lung metastasis via intratumoral or intravascular IL-12gene therapy	Murine or rat model of HCC (in vivo)	Cytokine gene therapy	[64, 85, 86]
Increasing the serum levels of IL-12 and activation of NK cells via transferring CD40L gene into dendritic cells by adenovirus	Rat HCC model (in vivo)	Gene therapy	[87]
Anti-tumor activity of type I and type III interferons and critical role of NK cells in their activity	BNL hepatoma model of HCC (in vivo)	Cytokine gene therapy	[88]
Dual functional therapy involving both gene therapy with the aim of inhibiting tumor growth and immune-stimulatory (particularly NK cells) by inducing type I IFN production for treatment of HBV and HCC	(In vitro and in vivo)	Cytokine gene therapy	[89–93]
Activation of NK cells and clearance of HBV via blocking the inhibitory receptor NKG2A	In vivo	mAb therapy	[94]
Regulatory role of T-cell Ig and ITIM domain (TIGIT) as an inhibitory receptor on NK cells in acute viral hepatitis and liver regeneration	(In vitro and in vivo)	mAb therapy	[95–97]
Increasing cytotoxic activity of NK cells against HCC via co-culture with K562-mb15-41BBL cell line, enhancing anti-HCC effects of sorafenib by adding NK cells to the culture of HCC cell lines and inhibiting cytotoxicity of NK cells via blocking NKG2D antibody	Cytotoxicity assays (in vitro) and Xenograft mice model (in vivo)	Adoptive transfer of activated NK cells and Chemo-immunotherapy	[98]
Role of androgen receptor (AR) on NK cell activity by altering IL-12A expression and the effect of sorafenib on enhancing IL-12A expression via suppressing AR signals. Better suppression of HCC via combining sorafenib with NK cells.	Cell cytotoxicity test (in vitro) and liver orthotopicxeno graft mice model (in vivo)	Chemo-immunotherapy	[99]
Increasing HCC tumor growth and lung metastasis in sorafenib-pretreated mice. Reducing the number of NK cells and inhibition of NK cell cytotoxicity against tumor cells and proliferation of NK92-MI cells by sorafenib	In vivo and in vitro	Chemo-immunotherapy	[100]

#### Clinical trials of NK cell based therapy for HCC patients

Clinical trials cell-based products are commonly classified into four phases. If the cell based therapeutic approach successfully passes through Phases I, II, and III, it will be approved for use in the general population of phase IV that is ‘post-approval’ phase. Clinical trial studies about treatment of HCC patients based on NK cells, alone or combined with other agents that have been registered in the site of https://clinicaltrials.gov/, are illustrated in Table 2. These researches are in various phases of clinical trials with different therapeutic approaches and in diverse doses and times of cell injection [101].

**Table 2:** Clinical studies of NK cell based immunotherapy in the HCC patients

Interventions	Design	Strategy (brief summary)	Recruitment status	Clinicaltrials.gov identifier
“Safety and effectiveness study of autologous natural killer and natural killer t cells on cancer” including HCC	Phase I	NK cell and NKT cell-based autologous adoptive immunotherapy	Suspended	NCT00909558
“Evaluate the efficacy and safety of MG4101 (ex vivo expanded allogeneic NK cell)”	Phase 2	NK cell transfer after curative liver resection on the patient with advanced HCC	Completed	NCT02008929
By using adoptive transfer of autologous NK cells to prevent recurrence of hepatocellular carcinoma after curative therapy	Phase 2	Adjuvant adoptive immune therapy using NK cell in patient undergone curative resection (RFA or operation)	Not yet recruiting	NCT02725996
Safety study of NK cells from sibship to treat the recurrence of HCC after liver transplantation	Phase 1	Conventional treatment and NK cell transfer	Recruiting	NCT02399735
A study of MG4101 (allogeneic natural killer cell) for intermediate-stage of hepatocellular carcinoma	Phase 2	Allogeneic natural killer cell transfer after transarterial chemoembolization	Recruiting	NCT02854839
Safety study of liver natural killer cell therapy for hepatoma liver transplantation (MIAMINK)	Phase 1	Liver transplantation and liver NK cell inoculation	Completed	NCT01147380
Recombinant vesicular stomatitis virus expressing IFN-β and probable exertion of anti-tumor activity of NK cells against adult HCC	Phase I	Cytokine therapy	Recruiting	NCT01628640

### Effects of NK cells and sorafenib on each other’s performance against HCC

In study performed by Kamiya et al. [99] on immunotherapy of HCC by activated NK cells, two approaches have been applied for the activation of NK cells; expansion of NK cells by co-culturing them with K562-mb15-41BBL and stimulation of these cells overnight with 1000 IU/mL IL2. The results of cytotoxicity assays against several HCC cell lines have illustrated that cytotoxicity of expanded NK cells were remarkably higher than that of the unstimulated or IL2–stimulated NK cells. Furthermore, treatment of immune-deficient Hep3B engrafted mice with expanded NK cells significantly improved overall survival rate and reduced tumor growth. In addition, their results from in vitro experiment on comparative cytotoxicity of sorafenib and NK cells have illustrated that NK cells dramatically increased the anti-HCC effects of sorafenib in HCC cell lines that were pre-exposed to 5 μM sorafenib for 48 h. On the other hand, the cytotoxicity of NK cell has appeared to be unaffected in the presence of sorafenib which suggests that the combination of sorafenib and NK cells could have beneficial effects on the treatment of HCC [99].

In another study conducted by Zhang et al. [101], it has been illustrated that pre-treatment of BALB/c nu/nu mice and C57BL/6 mice by sorafenib leads to accelerated tumor growth, decreased mouse survival and increased lung metastasis. They have suggested that the efficacy of sorafenib in the treatment of HCC patients may be enhanced by immunotherapeutic approaches aiming to activate NK cells. Their study concluded that sorafenib remarkably reduced the number of NK cells and inhibited their proliferation as well as anti-tumor function by blocking AKT and ERK phosphorylation pathways which might indicate the off-target effects of sorafenib [101, 102]. Indeed, as resulted in the work of Lohmeyer et al. [103] the effect of sorafenib on the NK cell effector functions was a time- and dose-dependent manner. They have showed that long-term pre-treatment of NK cells with sorafenib enhances their cytokine expression and degranulation marker expression against target cells via activation of CRAF and ERK1/2 phosphorylation in NK cells. They have also demonstrated that the stimulatory effects of sorafenib is related to alteration of the MAPK/ERK pathway but not AKT signaling pathway (Fig. 1), as confirmed by other researcher groups [102, 104].

Krusch et al. [105] have investigated the effect of sorafenib on NK Cell anti-tumor reactivity in vitro. Their results have indicated that sorafenib-induced inhibition of cytotoxicity and IFN-γ production of NK cells, when encountered with renal cell carcinoma cell lines, was due to impaired regulation of NK cell reactivity via PI3K and ERK phosphorylation. Therefore, considering multiple new approaches to combine protein kinase inhibitors treatment with immunotherapy, determining the effectiveness and optimal doses of PKI for cancer therapy requires further investigation [105]. Furthermore, activation of cAMP/PKA-dependent Raf/MEK/ERK signaling by sorafenib has been described in cholangiocytes [106]. So, there are two different approaches: on the one hand, RAF inhibitors such as sorafenib seem to be potent induction for the pre-activation of NK cells, on the other hand, in vitro pre-treatment with clinically relevant concentrations of sorafenib leads to impaired NK-cell effector functions [106] and higher doses of sorafenib might lead to immunosuppression and reduced anti-tumor response by NK cells in vivo [101, 107, 108]. So, a careful dose and time optimizing is necessary as high drug concentrations inhibit proliferation and activation of NK cells against tumor cells, while treatment with certain dosage levels for long-term can lead to activating effects on NK cell functions [103].

### Mechanism of correlation between sorafenib and NK cell in HCC treatment

**Fig. 2:**
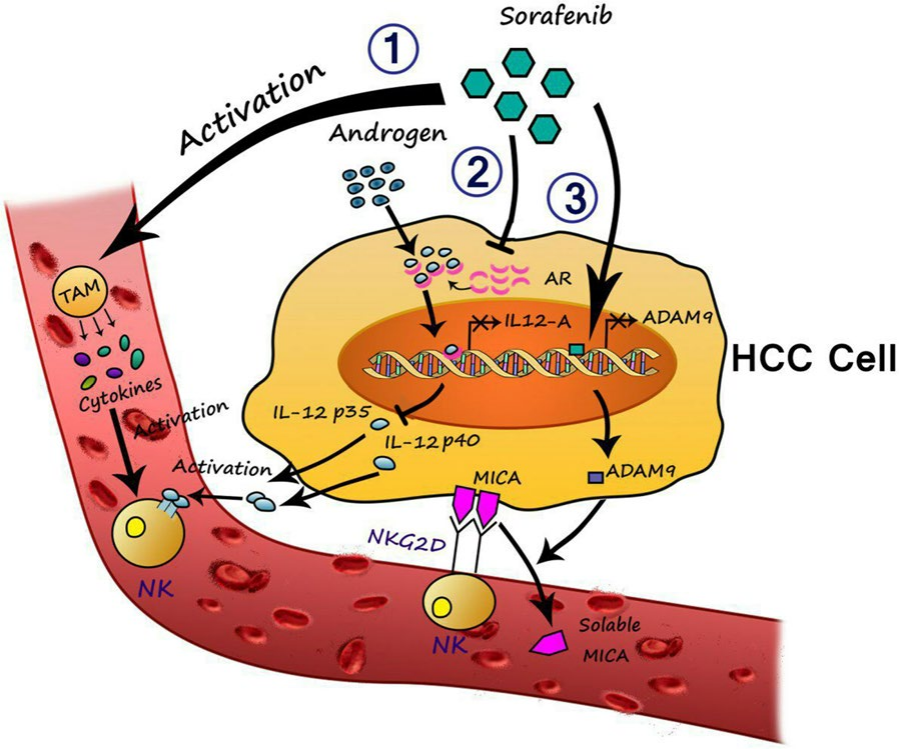
Three mechanisms of the effects of sorafenib on NK cell activation against HCC cells

Several studies have been recently done on the mechanisms of sorafenib plus NK cells efficacy for better suppression of HCC progression (Fig. 2). Sprinzl et al. [74] have noted that short-term implementation of sorafenib leads to increased cytolytic NK cell function against tumor cells via activation of tumor-associated macrophages (TAM). The results of this study have shown that sorafenib activated hepatic NK cells in mice via triggering pro-inflammatory cytokine production, such as IL6, TNF-α and IL12 in polarized MΦ [74].

NKG2D is one of the most important activating receptor expressed on the surface of NK cells [39, 109]. Major histocompatibility complex (MHC) class I–related chain A (MICA) is a ligand for NKG2D, which is frequently expressed on the surface of tumor cells. Moreover, MICA-NKG2D pathway is an important mechanism for activation of NK cells and enhancing their cytolytic activity and cytokine production against HCC [70, 71, 110]. Tumor cells can escape from NKG2D mediated immune surveillance by proteolytic cleavage of membrane bound MICA molecules and formation of soluble forms in sera of HCC patients [58, 60, 111]. It has been reported that disintegrin and metalloproteinase (ADAM) proteins play essential roles in MICA transformation from membrane-bound forms to soluble forms [112, 113]. Kohga et al. [73] have investigated the effect of sorafenib on expression of ADAM9 protein and its association with shedding of MICA on HCC cells. They have illustrated that ADAM9 was over-expressed in human HCC tissues which resulted in decreased expression of membrane-bound MICA, increased production of soluble MICA and reduction of NK sensitivity of human HCC cells. The most important finding of this study was that sorefenib reduced the ADAM9 expression level in HCC cells, which led to inhibition of MICA ectodomain shedding, down-regulation of soluble MICA and up-regulation of membrane-bound MICA expression and consequently resulted in enhanced sensitivity of HCC cells to NK cells. Therefore, they have concluded that the combinational therapy of anti-HCC molecular targeted drug and immunotherapeutic approach for activation and enhancement of NK cells might improve the treatment protocol of HCC patients [72, 73].

Other mechanism that has been raised about the correlation of sorafenib and NK cells against HCC cells is targeting androgen receptor (AR) signals. Considering the key role of gender disparity involving AR during initiation and progression of HCC and normal function of liver [114] and its effect on the suppression of HCC metastasis [115], the correlation of AR expression and NK cell function in HCC suppression seems to be important. Furthermore, Shi et al. [100] have investigated the effect of sorafenib on the expression of AR and IL-12A and their role in the activation of NK cell for better treatment of HCC. They have concluded that AR reduced the expression of IL-12A by binding to the IL-12A promoter which ultimately led to repression of NK cell cytotoxicity against HCC cells. Importantly, the results of the in vivo study performed by Shi et al. [100] in orthotopic HCC mice model have demonstrated that treatment with sorafenib could enhance the activation of NK cells by up-regulation of IL-12A expression via inhibition of AR signals. They have not only explained the mechanism of AR roles in the gender disparity of HCC but have also provided a new approach of combinational therapy applying sorafenib and NK cells [100].

## Conclusion

The majority of studies have shown that NK cells and sorafenib could enhance each other’s performances and compensated the deficiency of one another in the advanced stages of HCC. However, some studies have reported that sorafenib reduced the number of NK cells and inhibited their proliferation as well as reactivity against HCC cells. The paradoxical effect of this combination treatment is time- and dose-dependent. Therefore, careful dose and time optimization is necessary for development of a combinational immunotherapy for HCC. Furthermore, adaptive NK cell therapy or immunotherapeutic approaches activating NK cells along with sorafenib treatment may be a promising strategy to improve the therapeutic efficacy of HCC patients.

## Abbreviations

HCC: hepatocellular carcinoma
NK: natural killer
Raf: rapidly accelerated fibro sarcoma
VEGFR: vascular endothelial growth factor receptor
PDGFR: platelet-derived growth factor receptor
TACE: trans-catheter arterial chemoembolization
EGFR: epidermal growth factor receptor
mTOR: mammalian target of rapamycin
Met: mesenchymal-epithelial transition
PD-L1: programmed death-ligand 1
mAB: monoclonal antibody
IFN: interferon
TGF: transforming growth factor beta
IL: interleukin
KC: Kupffer Cells
TLR: Toll-like receptor
Tregs: T-regulatory cells
MHC: major histocompatibility complex
GVHD: graft versus host disease
GvL: graft vs. leukemia
TNF: tumor necrosis factor
TRAIL: TNF-related apoptosis-inducing ligand
ADCC: antibody dependent cellular cytotoxicity
MDSCs: myeloid-derived suppressor cells
MICA: MHC class I–related chain A
TAM: tumor-associated macrophages
ADAM: disintegrin and metalloproteinase
AR: androgen receptor
